# Comparison of Resonance Frequency Analysis and of Quantitative Ultrasound to Assess Dental Implant Osseointegration

**DOI:** 10.3390/s18051397

**Published:** 2018-05-02

**Authors:** Romain Vayron, Vu-Hieu Nguyen, Benoît Lecuelle, Hugues Albini Lomami, Jean-Paul Meningaud, Romain Bosc, Guillaume Haiat

**Affiliations:** 1 CNRS, Laboratoire Modélisation et Simulation Multi Echelle, MSME UMR CNRS 8208, 61, Avenue du Général de Gaulle, 94010 Créteil CEDEX, France; romain.vayron@u-pec.fr (R.V.); vu-hieu.nguyen@u-pec.fr (V.-H.N.); hugues.albinilomami@univ-paris-est.fr (H.A.L.); 2 Centre de Recherche BioMédicale, Ecole Nationale Vétérinaire d’Alfort, 7 Avenue du Général de Gaulle, 94700 Maisons-Alfort, France; benoit.lecuelle@vet-alfort.fr; 3 Service de Chirurgie Orthopédique et Traumatologique, Hôpital Henri Mondor AP-HP, CHU Paris 12, Université Paris-Est, 51 Avenue du Maréchal de Lattre de Tassigny, 94000 Créteil, France; meningaud@me.com (J.-P.M.); romainbosc@gmail.com (R.B.); 4 Équipe 10, Groupe 5, IMRB U955, INSERM/UPEC, 8 rue du Général Sarrail, 94000 Créteil, France

**Keywords:** dental implant, resonance frequency analysis, quantitative ultrasound, osseointegration, implant stability

## Abstract

Dental implants are widely used in the clinic. However, there remain risks of failure, which depend on the implant stability. The aim of this paper is to compare two methods based on resonance frequency analysis (RFA) and on quantitative ultrasound (QUS) and that aim at assessing implant stability. Eighty-one identical dental implants were inserted in the iliac crests of 11 sheep. The QUS and RFA measurements were realized after different healing times (0, 5, 7, and 15 weeks). The results obtained with the QUS (respectively RFA) method were significantly different when comparing two consecutive healing time for 97% (respectively, 18%) of the implants. The error made on the estimation of the healing time when analyzing the results obtained with the QUS technique was around 10 times lower than that made when using the RFA technique. The results corresponding to the dependence of the ISQ versus healing time were significantly different when comparing two directions of RFA measurement. The results show that the QUS method allows a more accurate determination of the evolution of dental implant stability when compared to the RFA method. This study paves the way towards the development of a medical device, thus providing a decision support system to dental surgeons.

## 1. Introduction

Dental implants [1] are now used in clinical routine and have allowed for important progress in maxillofacial and oral surgery. However, implant failures still occur and remain difficult to anticipate. Dental implant stability, which is determinant for the surgical success [2], is determined by the quantity and biomechanical quality of bone tissue around the implant [3]. Two kinds of implant stability may be distinguished. The primary stability occurs at the moment of implant surgical insertion within bone tissue. Dental implant primary stability should be sufficiently important in order to avoid excessive micromotion (higher than around 50 µm) at the bone-implant interface after surgery, but the pressure on the alveolar bone should not be too high in order to avoid bone necrosis that is related to bone tissue overloading [4]. Secondary stability is obtained through osseointegration process, a complex phenomenon of a multi-time and multiscale nature [4], which strongly depends on primary implant stability [5].

Dental implant stability remains difficult to be assessed clinically because it depends on the implant properties (geometry, surface properties …), on the patient characteristics and bone quality, as well as on the surgical protocol. There is a lack of standardization of the surgical procedures used in oral implantology. For example, the duration between implant insertion and loading is often chosen empirically by the surgeons and can vary from 0 up to 6 months [6]. A specific compromise should be found for each patient between (i) an early or even immediate [7] implant loading in order to enhance osseointegration phenomena, and (ii) a late implant loading in order to avoid degradation of the bone-implant interface [8]. Shortening the implant loading time has become a challenge in recent implant developments to (i) reduce the time of social disfigurement and (ii) avoid tissue loss. Therefore, the quantification of implant primary and secondary stability is of interest since it could be used to improve the surgical strategy by adjusting the choice of the healing time in a patient-specific manner.

Assessing the implant stability is a difficult multiscale problem because bone tissue is a complex medium and because of remodeling phenomena [9,10]. Different methods have been used to determine the implant stability in vivo. So far, most surgeons still rely on their proprioception [8] and accurate quantitative methods that are capable of quantifying implant stability, which are required to assist the surgeons and to eventually reduce implant failure risk.

Magnetic resonance imaging [11] and X-ray based [1] techniques remain of limited interest to measure implant stability because of diffraction phenomena occurring at the bone-implant interface due to the presence of metal. Therefore, mechanical methods have been developed, their advantage consisting in the absence of irradiation, portability and non-invasiveness. The measurement of the insertion torque to assess implant primary stability has been evoked but remains limited [12], because the result is not only related to the properties of the bone-implant interface and because it cannot be used for secondary stability assessment. The Periotest (Bensheim, Germany) consists in a percussion test method [13,14], but its sensitivity to striking height and handpiece angulation complicates the clinical examination [15] and limits the reproducibility of the measurements. The most widely used technique is the resonance frequency analysis (RFA) [16], which consists in measuring [17] the first bending resonance frequency of the implant. The Osstell© (Gothenburg Sweden) device is on the market and uses the RFA technique to measure the response of an implant through the implant stability quotient (ISQ). The RFA technique allows for assessing the implant anchorage depth into bone [18], marginal bone level [19], cortical bone thickness [20,21] and the stiffness of the bone-implant structure [22,23]. However, RFA cannot be used to identify directly the bone-implant interface characteristics [24]. No correlation between the implant stability quotient (ISQ) and bone implant contact (BIC) has been evidenced so far [25]. Different results are obtained when ISQ measurements are performed in different directions, which is indicated in the user manual of the Osstell device.

An alternative method to assess dental implant stability has been developed by our group and consists in using a quantitative ultrasound (QUS) method [26] to investigate the properties of the bone-implant interface. The principle of the measurement lies on the dependence of ultrasonic propagation within the implant on the boundary conditions that are given by the biomechanical properties of the bone-implant interface [27]. An in vitro preliminary study was carried out using a prototype titanium cylinder shaped implants that were inserted in bone tissue, showing the sensitivity of the ultrasonic response of the implant to the quantity of bone tissue that is in contact with the dental implant [27]. The principle of the measurement was then validated experimentally by showing the sensitivity of the ultrasonic response of a planar bone-implant interface to healing time with coin-shaped implant models [28]. Significant variations of the ultrasound response of dental implants that are embedded in a bone substitute biomaterial made of a tricalcium silicate-based cement was shown to occur when the implants are subjected to fatigue loading [29]. More recently, another in vitro study proved the potentiality of QUS methods to assess dental implant primary stability [30]. A preclinical validation of the device in rabbits was carried out [31], and showed that the measurement was significantly sensitive to healing time. More recently, the results that were obtained with RFA and QUS techniques were compared using implants that were inserted in bone mimicking phantoms [32]. Different values of trabecular bone density and cortical thickness were considered to assess the effect of bone quality on the respective indicators (UI and ISQ) [32]. The effects of the implant insertion depth and of the final drill diameter were also investigated. The results showed that the QUS technique provides a better estimation of different parameters related to the implant stability compared to the RFA technique. Moreover, finite difference [33] and finite element numerical simulations [34,35] were carried out to understand the interaction between an ultrasonic wave and the bone-implant system. However, the previous in vivo measurements were realized with an ultrasonic probe that was manually positioned on the implant abutment screw, leading to reproducibility issues [31]. Moreover, the behaviour of implants inserted in big animal models is still missing and would be of interest because bone properties are closer to those of human bone tissue. Eventually, the comparison of the in vivo results that were obtained using the two methods (QUS and RFA) would also be of interest in order to better assess the performance of the different approaches.

The aim of the present study is to compare two different methods (i.e., RFA and QUS approaches) that have been evoked to assess dental implant stability. A new version of the QUS probe that had not been investigated in vivo was considered herein. The objective is to compare the dependence of the results obtained with the RFA and QUS techniques as a function of healing time. To do so, our strategy consists in using dental implants inserted in sheep iliac crest bone because it corresponds to a big animal model having bone properties that are close to those of human bone tissue [36,37]. The measurements were realized with both techniques (RFA and QUS) at different healing times (0, 5, 7, and 15 weeks).

## 2. Materials and Methods

### 2.1. Implants

**Figure 1:**
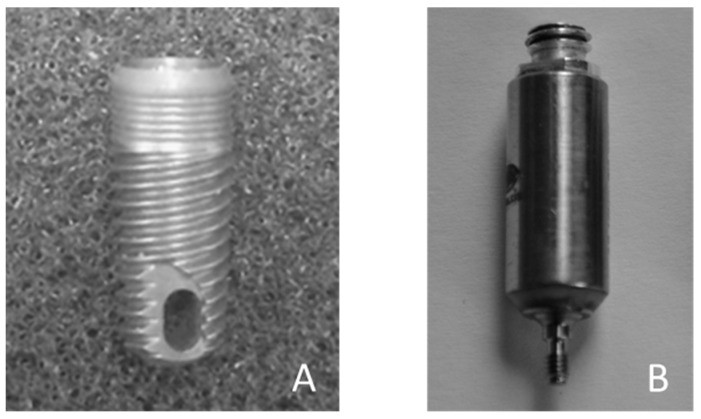
(**A**) Dental implant (Zimmer Biomet, TSVT4B10); (**B**) Planar ultrasonic contact transducers generating a 10 MHz broadband ultrasonic pulse.

Eighty-one identical TiAl6V4 titanium alloy, 10 mm long and 4 mm diameter conical dental implants were used in the present study. The implants were manufactured by Zimmer Biomet^®^ (Warshaw, IN, USA) under the reference TSVT4B10 (see Figure 1A).

### 2.2. Animal

Eleven 2–3 year-old female «préalpes» sheep (France) were used in this study. The animals were housed in a box in an environment (ambient temperature 19 °C and a humidity of 55%) in accordance with the requirements of The European Guidelines for Care and Use of Laboratory Animals. The study has been approved by the ethical committee of the Alfort National Veterinary School (ENVA). Artificial lighting and air conditioning systems were used in the animal housing facility. Commercial food was given to the animals and water has been provided ad libitum.

### 2.3. Surgical Procedure

The sheep were anesthetized via intravenous injection (IV) of 0.5 mg/kg Diazepan (Valium^®^, Roche, Basel, Switzerland), 5 mg/kg ketamine hydrochloride (Ketamine 1000^®^, Virbac, France). The intratracheal intubation was realized with a 8 mm endotracheal probe that was connected to a respirator in mode «ventilation volume controlled mode» by using isoflurane (between 2% and 2.5% in a combination air/oxygen (QSP EtO_2_ 50%). Prior to surgery, operating sites (iliac crest) were carefully shaved and were disinfected with a 10% chlorhexidine alcoholic solution and povidone-iodine solution. Before the surgery, injections of 0.1 mg/kg Morphin (IV, every 2 h), 0.4 mg/kg Meloxicam (intramusculous IM), and 0.1 mL/kg Shotapen (IM) were realized.

A 10 cm longitudinal skin incisions was made to expose the iliac crest of the sheep. An offset of 1 cm was considered relatively to the iliac crest to avoid friction of the implants on the operative scar. The lateral resection of these tissues exposed the underlying periosteum. A medial-anterior incision was made and the periosteum was elevated with a periosteal elevator and retained by a retractor, thus exposing the bone implantation site.

**Figure 2:**
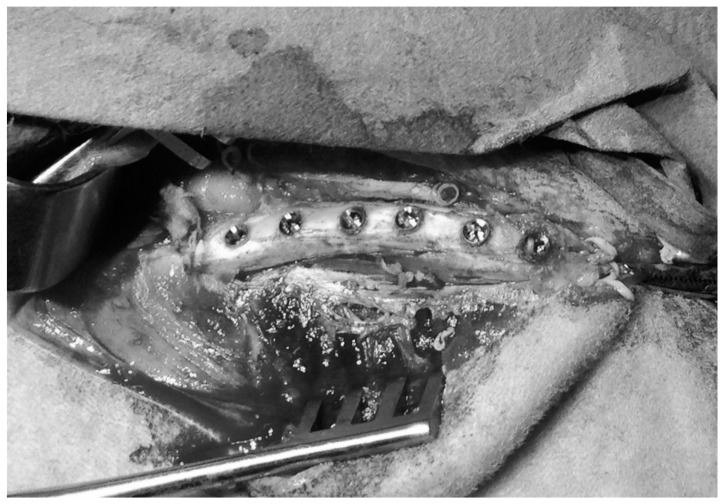
Photography of six dental implant placed in a sheep iliac crest.

As shown in Figure 2, between four and six dental implants (according to the available space) were placed in both iliac crests of each sheep, following the procedure described by Pearce et al. [36,38,39,40] and shown in Figure 2. The space available between the center of two neighboring implants was higher than 1 cm in all cases. Following the recommendation of the manufacturer, a conical cavity (10 mm deep and 3.4 mm wide) was created in the iliac crest in a step wise fashion, using color-coded, 10 mm-length surgical drills (2.3, 2.8, 3.4 mm diameter; Zimmer Biomet). All of the osteotomies were thoroughly rinsed with isotonic saline to remove bone fragments prior to the insertion of the titanium implants.

After the surgery, injections of 0.2 mg/kg Morphin (SC, every 24 h during three days), 0.4 mg/kg Meloxicam (IM, one time after 48 h) and 0.1 mL/kg Shotapen (IM, one time after 72 h) were realized. The sheep were euthanized at 15 weeks post first implantations by IV injection of 20 mL of Dolethal (pentobarbital 18%).

### 2.4. Experimental Protocol

#### 2.4.1. Quantitative Ultrasound Measurements

**Figure 3:**
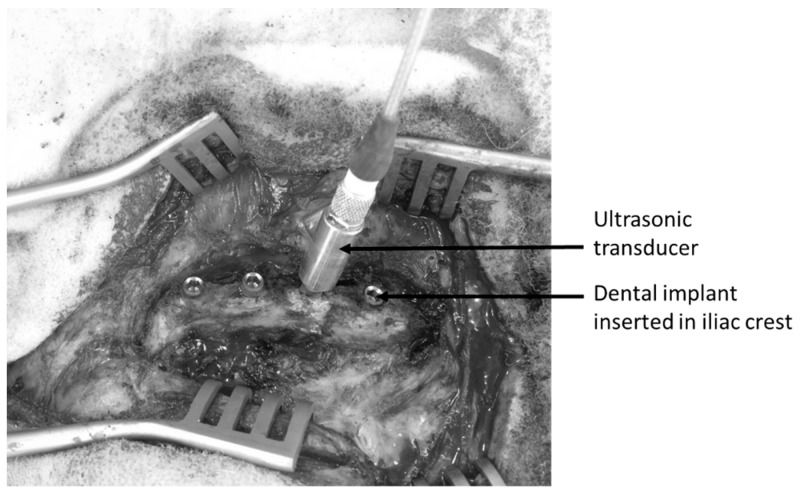
Measurement configuration of the ultrasonic indicator using the ultrasonic transducer screwed into a dental implant.

Figure 1B shows the dedicated QUS probe used herein that was manufactured by Sonaxis (Besançon, France). This probe is a monoelement transducer, including a 4 mm diameter planar ultrasonic contact transducers and generating a 10 MHz broadband ultrasonic pulse propagating perpendicularly to its active surface. The probe was attached rigidly to a titanium alloy dental healing abutment, which can be screwed into the implant so that the measurements are not influenced by positioning problems of the probe relative to the healing abutment, as it was the case in previous studies [29]. The QUS device was screwed into the implant in order to realize each measurement for each implant and each configuration, as shown in Figure 3. The ultrasonic probe was linked to a pulser-receiver via a standard coaxial cable. A transient recorder was used to record the radiofrequency (rf) signal with a sampling frequency equal to 100 MHz.

For each measurement, the transducer was screwed in the implant with a controlled torque that was equal to 3.5 N·cm, which is significantly lower than the torque recommended for the implant insertion [41], for the final prosthetic abutments and for the healing abutment. Therefore, screwing the transducer is not likely to induce degradation of the bone implant interface. Then, the echographic measurement was realized instantaneously. The transducer was then unscrewed and the same measurement was carried out a total number of three times in order to assess the reproducibility of the measurements.

The same signal processing technique as the one used in [29] was used to derive a quantitative ultrasonic indicator *I*, which had been shown to be related with dental implant stability [30,31]. The indicator *I* was devised to quantitatively estimate the average amplitude of the signal between 10 µs and 120 µs. The envelop *S*(*t*) of the *rf* signal s(t) was determined and the indicator *I* was given by:

(1) $$I=\sum_{i=1000}^{12,000}S\left(iT_{0}\right),$$

where *T*_0_ = 0.01 µs corresponds to the sampling period. The time window chosen to determine the indicator *I* was chosen, as follows. The upper bound of the time window was chosen equal to 120 µs, which corresponds to a compromise between a sufficient duration to obtain pertinent information and the requirement of a sufficient signal to noise ratio for all *rf* signals. Moreover, the time window that was used to compute the indicator I starts at a time of 20 µs because the amplitude of the envelop of the *rf* signals before 20 µs is approximately constant due to a saturation of the amplitude. In this study, we modified the ultrasonic indicator I that was used in previous studies [29,30,31] in order to obtain an ultrasonic indicator (UI) (i) having values comprised from 1 to 100 (similar to the indicator obtained with RFA device) and (ii) increasing when bone quality and quantity increases around the implant. The indicator *UI* was defined by:

(2) UI=100−10×I

The average and standard deviation values *UI_m_* and *UI_std_* of the indicator UI that was obtained for the three measurements realized to assess the reproducibility of the measurement were determined for each implant and each configuration.

#### 2.4.2. Resonnance Frequency Analysis Measurements

**Figure 4:**
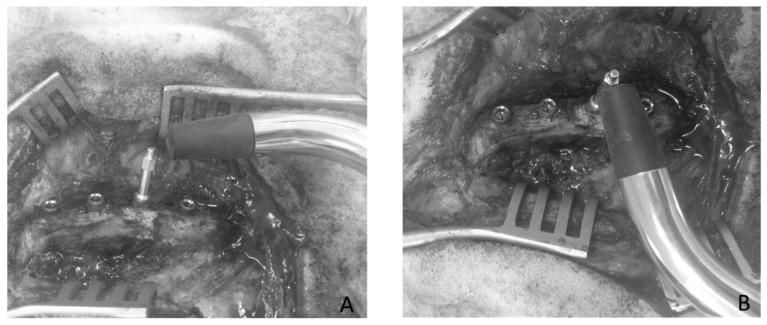
Measurement configuration of the implant stability quotient (ISQ) using the resonance frequency analysis (RFA) device realized in two perpendicular directions ((**A**) 0° and (**B**) 90°).

The RFA response of each implant in each configuration, was measured in *ISQ* units using the Osstell© device (Osstell, Göteborg, Sweden). Figure 4 shows the configuration of the measurements which were realized using the dedicated Smartpeg provided by the manufacturer and screwed into the implant, following the recommendation of the manufacturer. Each measurement was performed three times (in order to assess the reproducibility of the measurements) in two perpendicular directions, denoted 0° and 90°, relative to iliac crest direction. The ISQ values obtained in the 0° (respectively 90°) direction was noted *ISQ*0 (respectively *ISQ*90). The average and standard deviation values *ISQ_m_* and *ISQ_std_* of the three different ISQ values were determined for each implant and each configuration.

#### 2.4.3. Measurement Protocol

**Figure 5:**
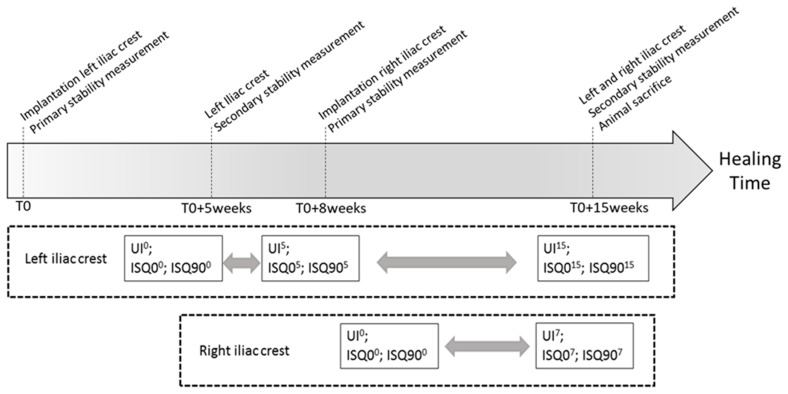
Schematic representation of the surgical protocol. The grey arrows indicate the comparison between the different healing times performed herein.

For each animal, the protocol summarized in Figure 5 was carried out. Firstly, dental implants were inserted in the left iliac crests following the protocol that is described in Section 2.3. Then, the QUS measurements were carried out three times and the RFA measurements were carried out three times for each direction. Five weeks later, the iliac crests were exposed again and the QUS and RFA measurements were realized in order to determine the variation of the UI and of the ISQ, respectively.

Eight weeks after the first surgery, dental implants were placed in the right iliac crests of all sheep, and QUS and RFA measurements were realized in order to determine the primary stability of each dental implant. Eventually, fifteen weeks after the first surgery, the UI and the ISQ were measured again for each implant in both iliac crests and the animals were euthanized.

We chose to implant the right iliac crest eight weeks after the first surgery (T0+8 weeks in Figure 5) and to sacrifice the animals 15 weeks after the first surgery (T0+15 weeks in Figure 5) in order to obtain two groups of implants with 7 and 15 weeks of healing time in the same animal, which allows for carrying out a quantitative comparison between the two values of healing time.

For each implant, the value of *UI_m_* (respectively, *ISQ_m_*) obtained for a given healing time equal to n weeks is denoted $UI_{m}^{n}$ (respectively, $ISQ_{m}^{n}$).

#### 2.4.4. Data Analysis

In order to investigate the relative variation of both indicators as a function of healing time, analyses of variance analyses (ANOVA) were performed for each implant to evaluate the effect of healing time on different indicators, such as *UI*, *ISQ*0, *ISQ*90, and *ISQ*. Statistical differences were defined at a 95% confidence level. ANOVA allows for determining whether healing time has a significant effect on the different parameters described above.

The variation of each indicator (ISQ and UI) as a function of healing time was investigated by defining the following variables, which correspond to relative variation of the indicators between two healing time (here *n* and *p* weeks, *p* > *n*) for the same implant:

(3) $$\Delta_{ISQ}^{n-p}=ISQ_{m}^{p}-ISQ_{m}^{n}$$

(4) $$\Delta_{UI}^{n-p}=UI_{m}^{p}-UI_{m}^{n}$$

In cases where ANOVA shows that the results obtained with the indicator *X* after *n* and *p* weeks of healing time are different, the sign of $\Delta_{X}^{n-p}$ allows to determine whether the parameter *X* increases as a function of healing time ($\Delta_{X}^{n-p}>0$) or whether it decreases ($\Delta_{X}^{n-p}<0$).

The results can also be analyzed in order to simply determine the sensitivity of the QUS and RFA techniques to healing time, similarly as what has been done in [30,32]. To do so, a simple two step method is described in what follows.

The first step was to perform a linear regression analysis of $UI_{m}^{n}$ and $ISQ_{m}^{n}$ as a function of healing time, which leads to the following approximated relations:

(5) $$\tilde{ISQ}=a_{ISQ}\;t+b_{ISQ}$$

(6) $$\tilde{UI}=a_{UI}\;t+b_{UI},$$

where *t* corresponds to healing time, $\tilde{ISQ}$ and $\tilde{UI}$ are the approximated values of ISQ and UI, respectively, and $a_{ISQ}$ and $b_{ISQ}$ (respectively, $a_{UI}$ and $b_{UI}$) are the coefficients found by applying a linear regression analysis to the variation of the ISQ (respectively, the UI) as a function of *t*.

The second step of the method consists in using the averaged reproducibility error (*ISQ_std_* and *UI_std_*) corresponding to a given configuration in combination with the linear regression analyses corresponding to Equations (7) and (8), in order to assess the error realized on the estimation of the healing time *t*, noted in what follows *t_ISQ_* (respectively *t_UI_*) for the error realized using the RFA (respectively, QUS) technique:

*t_ISQ_* = *ISQ_std_*/*a_ISQ_* (7)

*t_UI_* = *UI_std_*/*a_UI_* (8)

As expected, the error on the estimation of the healing time *t* increases when the reproducibility error (given by *ISQ_std_* and *UI_std_*) increases and when the sensitivity of the method (given by $a_{ISQ}$ and $a_{UI}$) decreases in absolute value.

## 3. Results

### 3.1. Results for One Given Implant

**Figure 6:**
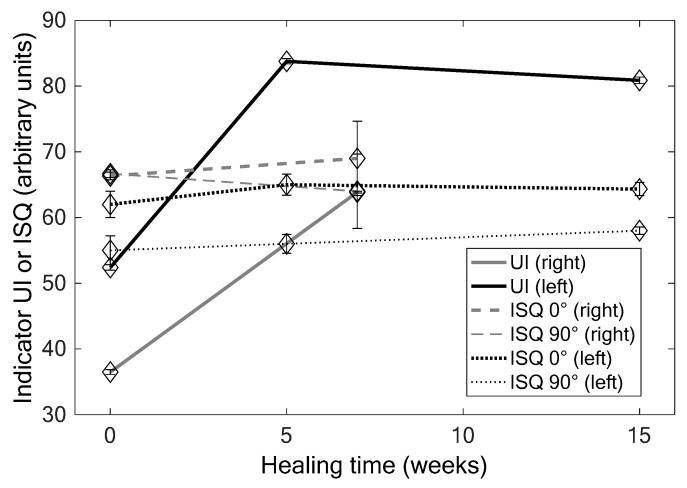
Results obtained for implant #2 right and left of the sheep #3 for the different healing times for *UI*, *ISQ* 0° and *ISQ* 90° values.

Figure 6 shows the results that were obtained for the implant #2 of the sheep #3 with the QUS and the RFA techniques. ANOVA shows that the values of the UI significantly vary as a function of healing time (0, 5 and 15 weeks: *p* = 6 × 10^−13^, *F* = 31; 0 and 7 weeks: *p* = 2.09 × 10^−9^, *F* = 5 × 10^4^), while the results that were obtained with the RFA technique do not depend on healing time (0, 5 and 15 weeks: *p* = 0.19, *F* = 4; 0 and 7 weeks: *p* = 1, *F* = 0). Note that the values of *ISQ*0 significantly increase as a function of healing time, while the opposite behaviour is obtained for *ISQ*90 (data not shown). As shown in Figure 6, the errors realized on the estimation of healing time are significantly lower when using the QUS device compared to the RFA device.

Table 1 shows the corresponding values of Δ*_X_*, *a_X_* and *t_X_* obtained for each indicator *X* and each couple of healing time. The results show the error made on the estimation of healing time is significantly lower when using the QUS device compared to the RFA device.

### 3.2. Results Obtained When Pooling All Implants

When pooling all QUS data obtained at 0, 5, and 15 weeks of healing time together (left iliac crest), ANOVA shows a significant effect of healing time on the indicator UI (*p* = 1.5 × 10^−11^ and *F* = 30.99). A similar result was obtained when pooling all QUS data at 0 and 7 weeks of healing time (*p* = 4.1 × 10^−8^ and *F* = 36.3). However, ANOVA analysis shows that there was no effect of healing time on the ISQ values when pooling all data obtained at 0, 5, and 15 weeks of healing time together (*p*-value = 0.50 and *F*-statistic = 0.68) and when pooling all data obtained at 0 and 7 weeks of healing time together (*p*-value = 0.35 and *F*-statistic = 0.98). Note that similar results were obtained when considering the variations of *ISQ*0 and *ISQ*90, respectively (data not shown).

Table 2 summarizes the different results obtained for the ISQ and UI measurements for all data pooled. Table 2 indicates the number of implants for which (i) the ANOVA analysis indicates that no significant difference was obtained, (ii) the indicator increases significantly as a function of healing time and (iii) the indicator decreases significantly as a function of healing time.

When comparing the results that were obtained after 0 and 5 weeks of healing time, no significant difference of *ISQ* values was obtained for most implants (24 out of 38), while a significant difference was obtained for UI values for all of the samples except one. Namely, a significant increase of the UI was obtained between 0 weeks and 5 weeks for 36 dental implants out of 38, which corresponds to an increase of the implant stability. These results are consistent with the mean and standard deviation values obtained for $\Delta_{UI}^{0-5}$ and $\Delta_{ISQ}^{0-5}$ shown in Table 2.

Similar results are obtained when comparing the results obtained after 0 and 7 weeks of healing time. No significant difference of *ISQ* values was obtained for most implants (24 out of 38), while a significant difference was obtained for UI values for all of the implants. Namely, a significant increase of the UI was obtained between 0 weeks and 7 weeks for 37 dental implants out of 43, which corresponds to an increase of the implant stability. These results are consistent with the mean and standard deviation values obtained for $\Delta_{UI}^{0-7}$ and $\Delta_{ISQ}^{0-7}$ shown in Table 2.

Again, when comparing the results obtained after 5 and 15 weeks of healing time, no significant difference of ISQ values was obtained for most implants (16 out of 29), while a significant difference was obtained for UI values for all samples except two. A significant increase (respectively decrease) of the UI was obtained between 5 weeks and 15 weeks for 11 (respectively, 16) dental implants out of 29. Moreover, a significant increase (respectively decrease) of the ISQ was obtained between 5 weeks and 15 weeks for 10 (respectively 3) dental implants out of 29. These results indicate that the results obtained depend on the dental implants considered. Note that 38 implants were considered between 0 and 5 weeks of healing time while only 29 implants were considered between 5 weeks and 15 weeks of healing time, which is due to the fact that nine implants were lost between 5 weeks and 15 weeks.

Table 3 summarizes the results obtained for the ISQ measurements in both directions (*ISQ_m_*). Table 3 indicates the number of implants for which (i) the ANOVA analysis indicates that no significant difference of *ISQ_m_* was obtained, (ii) *ISQ_m_* increases significantly as a function of healing time and (iii) *ISQ_m_* decreases significantly as a function of healing time.

### 3.3. Temporal Sensitivity

Table 4 shows the error realized on the estimation of the healing time, which is expressed by *t_ISQ_* (respectively, *t_UI_*) for ISQ (respectively, UI) measurements. As shown in Table 4, the error realized using the ultrasound device is always significantly lower compared to that realized using RFA measurements.

Table 5 shows the number of implants for which the error on the estimation of healing time is minimal when considering each indicator. The QUS technique is more accurate than the RFA technique for 104 out of a total of 1100 cases, which indicates a better precision of the QUS technique when compared to the RFA.

## 4. Discussion

To the best of our knowledge, this study constitutes the first attempt to investigate the dependence of the results that were obtained with the QUS and RFA technique as a function of healing time. Previously, the 1 MHz response of a screw inserted in aluminium was measured [26] and the approach has been extended to cylindrical implant models inserted in bone tissue [27] using 10 MHz transducers. The results have been explained using finite difference time domain numerical simulation tools [33]. More recently, the ultrasonic response of dental implants that were embedded in tricalcium silicate based cements and subjected to fatigue loading has been measured [29]. Another study that was aimed at assessing the variations of the ultrasonic response of a dental implant as a function of the insertion depth, which is related to its primary stability [30]. In a more recently study [32], we have compared the RFA and QUS techniques in order to investigate the primary stability of dental implants that were inserted in artificial bone blocks manufactured by OrthoBones^®^ (3Bscientific, Hamburg, Germany). Different stability conditions were considered by varying parameters, such as the type of bone block (trabecular bone density, cortical thickness), the final diameter drill and the insertion depth, in order to simulate different situations mimicking the variations of dental implant primary stability. Using the same method as the one described in Section 2.4.4, we also found that the ultrasound technique allowed for more precise estimations of the parameters that were varied in order to modify the primary implant stability. These results are in agreement with the results that were obtained in Table 4 and Table 5, which can be explained by a better reproducibility and a better sensitivity of the QUS method compared to the RFA method.

Another explanation of the better sensitivity of the QUS technique when compared to the RFA technique may be related to the principle of the measurement itself. The ISQ is related to the measurement of the resonance frequency of the bone-implant system, which depends not only on the bone-implant interface, but also on properties of the entire host bone that vibrates when excited mechanically. However, in QUS techniques, previous in silico studies [35] have shown that the amplitude of the echographic response of the dental implant is related to bone properties at a distance of around 30 µm around the implant, which is precisely a region where osseointegration phenomena are known to occur. Therefore, the QUS technique is likely to have a better sensitivity to changes due to osseointegration, as shown in the present study.

In previous papers by our group [30,32,33,34,35], the ultrasound indicator *I* was shown to decrease when (i) trabecular density increases; (ii) cortical thickness increases; (iii) the final diameter drill decreases; and, (iv) the insertion depth increases. These results are in agreement with a previous in vivo paper by our group that showed that the indicator *I* decreases as a function of healing time [42]. The aforementioned results can be explained by the fact that the UI is related to the amplitude of the echographic response of the implant, which depends on the boundary conditions that are applied to the implant external surface. The implant acts as a wave guide in which the acoustical energy is trapped [33,34,35].

More specifically, the increase of the UI as a function of healing time obtained between 0 weeks and 5 weeks and between 0 weeks and 7 weeks (see Table 2) can be explained by the association of two phenomena. First, the quantity of bone tissue in contact with the implant (denoted in the literature Bone-Implant contact, BIC) is known to increase versus healing time [43,44,45,46,47]. In the case of a debonded bone-implant interface, the implant is in contact with fibrous tissue and a stronger gap of mechanical properties is obtained at the implant surface, thus explaining that the transmission coefficient at the implant external interface is weaker [28,42] than when the bone tissue is in close contact with the dental implant. Consequently, energy leakage of the ultrasonic wave out of the implant, which acts as a wave guide [30,33], is lower when the bone-implant interface is debonded, which explains the higher ultrasonic energy that was recorded by the ultrasonic transducer. The acoustic energy recorded at the upper surface of the implant is lower when the implant external interface is in contact with bone tissue than when it is in contact with blood or fibrous tissue. Second, the mechanical properties of newly formed bone tissue around implants, such as the apparent Young’s modulus [48], the hardness [49], the ultrasonic velocity [50] and mass density [42], are known to increase versus healing time, which may be explained by the bone tissue remodeling and mineralization process. This increase of biomechanical properties according to healing time leads to a weaker impedance gap between the implant and the bone tissue, leading to higher ultrasound energy leakage. In summary, the combination of the BIC increase and of the bone biomechanical properties induces to a cumulative effect, leading a decrease of the reflection coefficient at the bone-implant interface versus healing time, and thus to a significant increase of the indicator UI as a function of healing time.

The main limitation of this study lies in the choice of the animal model and of the implantation area, which had already been used in the literature [36,38,39,40]. The interest of this animal model lies in the fact that many implants can be inserted in one animal, which is required by ethical considerations. Moreover, the use of big animal models allows for obtaining bone tissue that is closer to that of human bone tissue compared to rabbits. However, a strong limitation of the present animal model lies in the lack of mechanical stimulation applied to the implant. While a consistent increase of the UI was obtained for relatively low values of healing time (0–5 weeks and 0–7 weeks), the results that were obtained for higher healing duration (5–15 weeks) depend on the dental implant. These results may be explained by the fact the osseointegration may be triggered by wound healing event due to the cavity drilling, which stimulate bone remodeling phenomena after the implant insertion. However, dental implants implanted in sheep iliac crest are not loaded, which may explain possible bone loss after a certain amount of healing time. The UI decreases for most implants in the 5–15 time period, because the implants are not loaded mechanically, which is likely to lead to bone tissue resorption around the implants [51,52,53], thus explaining the decrease of UI. Note that similar results with a saturation of osseointegration after around 6 weeks were also obtained with implants inserted in Labrador dogs [25]. However, further studies should be realized in order to gain further insight on this issue.

Another limitation lies in the fact that only one implant type was used in this study, because the goal was to investigate the effect of variations of the implant stability on the UI and on the ISQ. It would be of interest to carry out the same study with other implant types. However, the QUS device was already validated using other implant types in previous studies [29,31].

The three healing times considered were chosen based on empirical considerations, which constitutes another limitation of the study. We choose the values of 5, 7 and 15 weeks of healing time based on previous publications [25] that considered comparable values. The choice of five weeks of healing time was chosen as a compromise between a sufficiently short time to obtain an early measurement and a sufficiently long time to be able to perform additional surgery after the surgical site healing (ethical requirement). The choice of seven weeks was chosen based on typical intermediate healing time found in the literature. The time of 15 weeks was chosen to obtain a relatively long healing time. The re-entry at five weeks also constitutes a limitation and is likely to possibly induce degradation of the implant stability as well as implant losses. Note that 9 implants were lost after five weeks, which may partly be due to the re-entry at five weeks. However, we made sure that the implant was not overloaded during the surgery at 5 weeks, since screwing the transducer with a torque of 3.5 N·cm correspond to low stresses that were applied to the bone-implant interface, which are not likely to induce implant failure.

This study showed that the QUS technique provides a better estimation of the implant stability compared to RFA, but the precise definition of a stable or unstable remains a difficult task [2]. The question of a target value of the UI remains an open problem that is not addressed in the present study since only comparisons are carried out. To answer this problem, a prospective clinical study would be needed, which is a perspective of the present work. Note that for ethical reasons, it was mandatory to realize pre-clinical studies before clinical ones.

## 5. Conclusions

This study allows to compare the results obtained with two different approaches (QUS and RFA methods), which were aiming at estimating primary and secondary dental implant stability, which are realized with the same implant model and with various healing times. All results are consistent and can be explained by physical analyses of the biomechanical phenomena occurring in and around the implant. Moreover, we found that the QUS technique leads to more significant variations of the indicator as a function of healing time when compared to the RFA technique. In particular, the error realized on the estimation of healing time using the QUS technique is around 10 times lower when compared to the RFA technique, which may be explained by the better reproducibility of the QUS measurements, as well as by the principle of the measurements. The present study paves the way for the development of an ultrasonic device to estimate dental implant stability that could be used in the clinic provided further in vivo buccal investigations.

## Figures and Tables

**Table 1:** Results obtained for implant #2 of the sheep #3. The values of the different indicators that are shown in Figure 6 allow for determining the variations $\Delta_{X}^{n-p}$, slopes *a_X_*, and sensitivity *t_X_* of both indicators for each healing times.

Healing Times *p*–*n*	Indicator *X*	Variation $\Delta_{X}^{n-p}$	Slope *a_X_*	Temporal Sensitivity *t_X_*
0–5	*UI*	31.4	6.3	0.2
	*ISQ*	2	0.4	9.2
5–15	*UI*	−2.9	−0.29	1.37
	*ISQ*	0.67	0.07	20.6
0–7	*UI*	27.4	3.9	0.24
	*ISQ*	0	0	∞

**Table 2:** Results obtained for the variation of the ultrasonic indicator (*UI*) and of the *ISQ* when comparing different healing times for all implants considered herein. $\Delta_{X}^{n-p}$ denotes the average difference of the results obtained for the indicator *X* between *p* and *n* weeks of healing time.

Healing Times *p*–*n*	0–5		5–15		0–7	
Indicator *X*	*UI*	*ISQ*	*UI*	*ISQ*	*UI*	*ISQ*
Mean $\Delta_{X}^{n-p}$	23.98	−0.9	−3.23	−2.4	20.47	1.9
Std $\Delta_{X}^{n-p}$	14.89	6.6	15.58	6.3	18.63	6.9
Total number of implants	38		29		43	
Nb of implants with a significant increase of *X*	36	5	11	10	37	8
Nb of implants with a significant decrease of *X*	1	9	16	3	6	16
Nb of implants with similar values of *X*	1	24	2	16	0	19

**Table 3:** Comparison of the results obtained for the variation of *ISQ* values between different healing times for the two direction of measurement (0° and 90°). The number of implants for which significant increase and decrease of *ISQ* was indicated, as well as the number of implants for which no significant difference was obtained for the two healing times considered.

Healing Times *p*–*n*	0–5		5–15		0–7	
Measurement direction	0°	90°	0°	90°	0°	90°
Total number of implants	38		29		43	
Nb of implants with a significant *ISQm* increase	1	12	5	9	2	8
Nb of implants with a significant *ISQm* decrease	7	7	9	2	10	13
Nb of implants with similar *ISQm* values	30	19	15	18	31	22

**Table 4:** Comparison of the error realized on the estimation of the healing time using the quantitative ultrasound (QUS) and RFA measurements in both directions (0° and 90°) and for both directions pooled. The mean and standard deviation values are shown as well as the maximum and minimum values for all implants and each time interval.

Healing Times *n–p*	Values	*t_UI_*	*t_ISQ_* for 0°	*t_ISQ_* for 90°	*t_ISQ_* Pooled
0–5	Mean ± Std	0.56 ± 2.04	4.59 ± 3.21	4.93 ± 4.56	9.58 ± 11.25
	Min/Max	0.07–12.7	0.52–9.89	0.6–14.4	0.74–57.6
5–15	Mean ± Std	1.28 ± 1.03	9.24 ± 11.9	13.93 ± 16.7	20.42 ± 31.8
	Min/Max	0.24–3.8	0.97–59.31	1.15–86.52	1.27 ± 172.8
0–7	Mean ± Std	0.90 ± 1.38	4.45 ± 5.33	9.67 ± 13.2	22.89 ± 39.4
	Min/Max	0.09–6.17	0.33–17.64	0.73–75.6	1.29–151.2

**Table 5:** Number of implants for which the estimation of healing time is minimal for the corresponding indicator and the corresponding healing period.

Healing Times *n–p*	*UI*	*ISQ*0	*ISQ*90	*ISQ* Both Directions Pooled
0–5	36	1	1	0
5–15	28	1	0	0
0–7	40	1	2	0
